# Haptic feedback intervention decreases the spatial margin when older adults walk through a narrow space

**DOI:** 10.1186/s40101-022-00315-y

**Published:** 2022-12-13

**Authors:** T. Hakamata, D. Muroi, K. Kodama, Y. Kondo, T. Higuchi

**Affiliations:** 1 Department of Health Promotion Science, Tokyo Metropolitan University, Tokyo, Japan; 2 Department of Rehabilitation, Kasai Central Hospital, Tokyo, Japan; 3 Chiba Prefectural University of Health Sciences, Chiba, Japan; 4 University Education Center, Tokyo Metropolitan University, Tokyo, Japan; 5 Tokushima Bunri University, Tokushima, Japan

**Keywords:** Obstacle avoidance, Aperture passing, Haptic feedback, Fingertip contact, Error learning

## Abstract

**Background:**

The ability to avoid obstacles efficiently and safely is important for older adults to prevent injuries from tripping and falling. It is important to find an optimal spatial margin between the body and an obstacle considering both safety and efficiency. One side of finding the optimal margin is to decrease the margin in terms of motor efficiency. In this study, we tested whether fingertip-contact intervention to obtain haptic feedback information to perceive the relationship between body and the environment could immediately improve spatial perception and collision avoidance behavior (an instantaneous effect).

**Methods:**

Twenty-seven older adults (12 males and 15 females) participated in the experiment. In the intervention of the fingertip-contact group, they lightly touched the edge of a door with both fingertips while walking. The test task before and after the intervention involved grasping a horizontal bar and passing through a narrow opening. As dependent variables, we measured the spatial margin and the collision rate.

**Results:**

The fingertip-contact group showed a significant decrease in the spatial margin after the intervention. On the other hand, there was no significant improvement in the collision rate after the intervention but rather a decrease only in the control group.

**Conclusion:**

The results obtained in this study indicate that touching obstacles with the fingertips had an instantaneous effect, leading to efficient movement learning, although a possible side effect of an increased collision rated was also found. The proposed intervention might promote an efficiency-based strategy due to learning the spatial relationship between the body and the environment, and it may suppress the excessive avoidance of older adults.

## Background

The modification of gait patterns in response to environmental constraints is referred to as adaptive locomotor adjustment [1, 2]. Due to aging-related changes in physical and perceptual/cognitive functions, the characteristics of adaptive locomotor adjustments in older adults are likely to be different from those in younger adults. This could lead to collision with an obstacle with insufficient and delayed modification [3–5] or destabilization as a result of avoiding the obstacle in an excessive manner [6, 7]. Finding a way to improve collision-avoidance behavior provides the requisite information for predicting collision-induced falls and injuries in older adults. Several studies have reported that repeated collision-avoidance experiences in a real environment [8] or virtual environment [9–11] improved participants’ obstacle-avoidance behavior [9, 10] or general mobility/stability [8, 11, 12]. Repeated experiences also improved their perceptual judgment regarding the passability of a narrow doorway [13], which could lead to safe navigation without collision. These studies suggest that obstacle-avoidance behavior improves with “learning by doing” [13].

Improvement of obstacle-avoidance behavior can be assessed from the viewpoints of two different strategies. The first strategy focuses on increasing the size of the spatial margin people keep between the obstacle and their body: a safety-based strategy. This strategy enables people to decrease their collision risk because they can keep a large spatial margin. The safety-based strategy, however, can lead to balance instability because people must change their posture dynamically during locomotion to keep too much margin [7]. For example, in the aperture-passing task, they must rotate their body if they keep the margin large. Rotating too much can cause balance instability and increase the fall risk.

A second strategy focuses on keeping the optimal spatial margin—an efficiency-based strategy. This strategy enables people to decrease their fall risk due to the abovementioned balance instability and to increase energy efficiency because they do not need to move largely. The efficiency-based strategy, however, can lead to an increased collision risk because people must be close to the obstacle when passing the aperture to keep the margin small. Thus, it is important to find and keep an optimal margin considering both safety and efficiency in obstacle-avoidance tasks. Finding such an optimal spatial margin is not simple, considering that the optimal relationship between the body and the environment should change depending on constraints of the performer, the environment, and the task [14, 15].

The intervention for the efficiency-based strategy, which enables participants to keep an optimal spatial margin, remains unknown. Considering that the safety-based strategy can lead to balance instability [7], it is worth seeking an intervention method to improve obstacle-avoidance efficiency. To do so, we assumed that actually touching the obstacle can increase the perception of the spatial relationship between the body and the environment. The theoretical backgrounds upon which we decided to test the effect of actually touching the obstacle are as follows. There are two approaches to motor learning—reductive and emergent views [16]. The reductive approach focuses on body functions and reduces them to representations and computations in the internal model [16–18]. From this view, fingertip-contact intervention is reasonable since the input of error signals (i.e., haptic information when touching the obstacle) is essential for learning and selecting modules in motor learning theory based on an internal model. On the other hand, the emergent approach focuses on task- or goal-specific organization or the emergence of an action system through a perception–action cycle within the environment [15, 16]. Behavior in the system is flexibly organized depending on the task or goal through interaction among the body, brain, and environment. Such fine-tuning of the task is called calibration [16, 19]. From the emergent view, fingertip-contact intervention is supposed to promote a perception–action cycle for learning the body–environment relationship and calibrating the system.

Based on these theoretical backgrounds, we hypothesized that the actual touching of the obstacle can increase perception of the spatial relationship between the body and the environment; as a result, the margin decreases, and the participant can acquire an efficiency-based strategy. To examine this hypothesis, we selected a walking-through-an-aperture task in accordance with previous studies [13, 20, 21]. We required participants to hold a horizontal bar [22, 23] in order to make the task relatively difficult, since it was hard for participants to improve their performance if the task was too simple.

## Materials and methods

### Participants

Twenty-seven older individuals (12 males and 15 females, mean age = 73.8 years, *SD* = 4.8 years) participated in the experiment. All participants had normal or corrected-to-normal vision. Their mean standing height was 159.6 cm (*SD* = 9.4 cm), and their mean body width at the shoulders, defined as the distance between the heads of the right and left humeri, was 40.3 cm (*SD* = 3.8 cm). Testing was approved by the Ethics Committee of Tokyo Metropolitan University, Japan (H29-8). Written informed consent was obtained from all participants in accordance with the Ethics Committee of Tokyo Metropolitan University and the Declaration of Helsinki. Participants received a bookstore gift card prior to participating in the experiment.

### Apparatus

The experiment was conducted in a room measuring 6.7 m × 4.9 m. The experiment was performed along a straight 5.5-m path. A custom-made, moving-door apparatus (Uchida Electronics Co., Ltd., Japan) was used to present an aperture. The apparatus consisted of an aluminum frame (2.4 m wide and 2.3 m tall) and two boards (0.6 m wide and 1.75 m tall). An aperture was created as the space between the two boards. Participants were to touch locations indicated by red sticky notes (2.5 cm wide and 7.5 cm tall) attached to the doors on both sides. The height of the note placement was set for each participant so that their upper limbs were naturally abducted when touching the notes.

In pre- and posttests, participants walked while holding a horizontal bar (1.5 cm in diameter). Holding a bar creates a task that requires more detailed perceptual readjustment. The bar was twice the participant’s shoulder width (distance between two acromia). Twelve three-dimensional motion-analysis cameras (OQUS300, manufactured by Qualisys, Sweden) were used to analyze the walking motion. The sampling frequency of the motion analysis was 120 Hz. The cameras tracked a total of 16 reflection markers. Three of these markers were attached to the frame of overhead headphones (Audio–Technica) to track the head movement (one at the top and one at each of the bilateral heads). Seven markers were attached to the participant’s body: one at each shoulder (lateral sides of the acromia), one at the intersection of the straight line connecting the lower shoulder blades and the spine, one on each side of the posterior superior iliac spine, and one on each index fingernail. Three markers were placed on each short board in a noncollinear arrangement to represent the board as a rigid body.

### Tasks and procedures

**Fig. 1:**
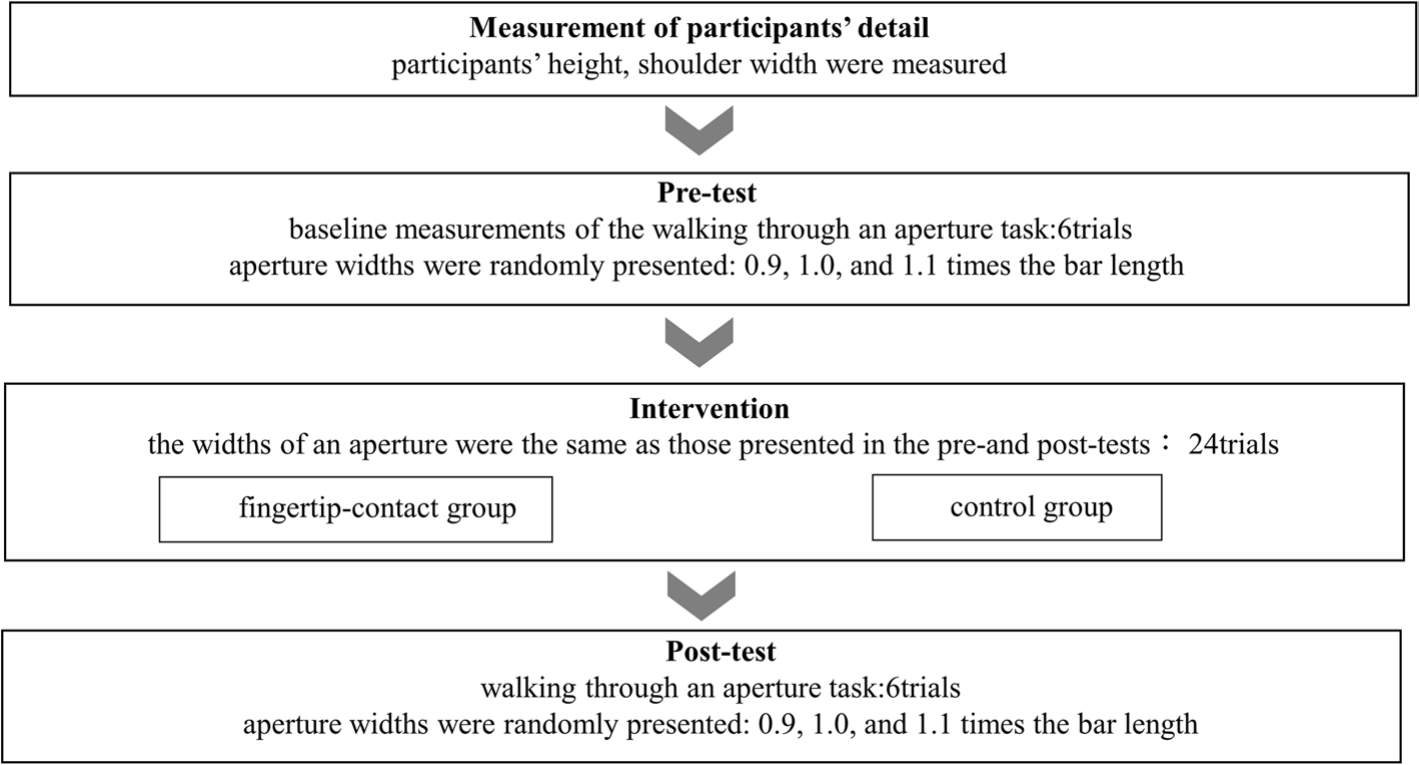
Flow chart of the procedure

The inclusion criterion was age ≥ 65 years, and participants were randomly assigned to one of two intervention groups: fingertip contact (*n* = 14) or control (*n* = 13). The experiment consisted of four parts (Fig. 1): (a) measurements of participants, (b) baseline measurements of the walking-through-an-aperture task (pretest), (c) intervention, and (d) post-test measurement of the walking-through-an-aperture task (posttest).

### Participants’ details

Participants’ height and body width at their shoulders were measured in cm. We also measured participants’ cognitive and mobility functions. Cognitive function was assessed using the Mini-Mental State Examination (MMSE). The MMSE is an 11-question test that measures five areas of cognitive function, with the maximum score being 30 [24]. A score of 23 or lower is indicative of cognitive impairment. Mobility function was assessed with the Timed Up and Go (TUG) test [25]. During the TUG test, participants were instructed to stand up from a standard chair with a seat height of 40 cm, walk for 3 m at a comfortable pace, turn, walk back to the chair, and sit down. The time required from the verbal command to begin the task to sitting down was measured with a stopwatch. Each participant performed the TUG task two times around the right and two times around the left; the average of these four times (in seconds) was used as the TUG score.

### Pre- and posttests

**Fig. 2:**
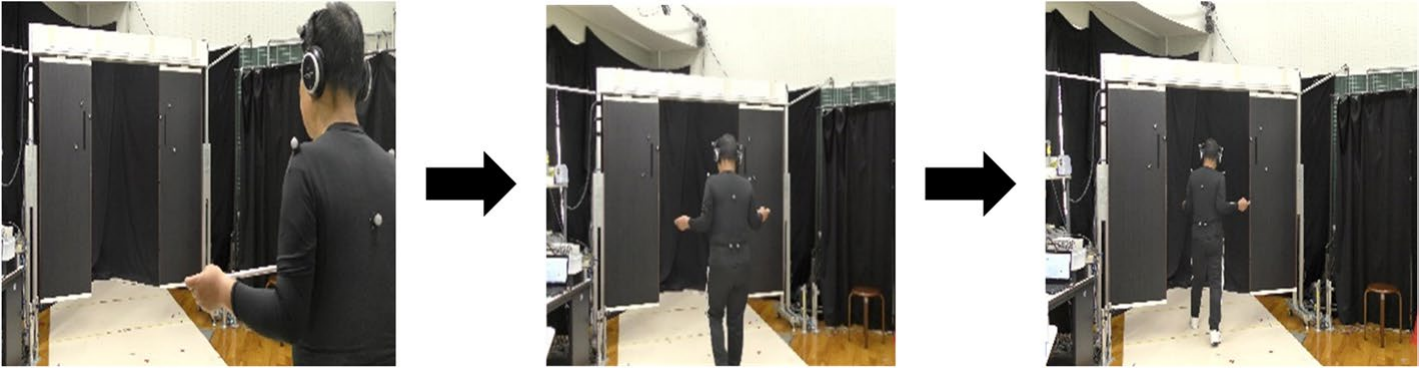
Method of gripping horizontal bars in pre- and posttests

Participants held both ends of a horizontal bar with both index fingers and fixed them at chest-high level (Fig. 2). This is a posture similar to that during the fingertip-contact intervention described later. Participants then walked toward each aperture from a distance of 4 m and tried to cross the aperture without collision. They were asked to do so while trying to minimize the spatial margin created between the bar and the doors. These instructions required more fine-tuning of their behavior. Three different aperture widths were randomly presented: 0.9, 1.0, and 1.1 times the bar length. In total, participants performed six main trials for each test (three aperture widths × two trials). Three practice trials (three aperture widths × one trial) were performed prior to the main trials in the pre-test session so participants could understand and familiarize themselves with the experimental procedure.

### Intervention

**Fig. 3:**
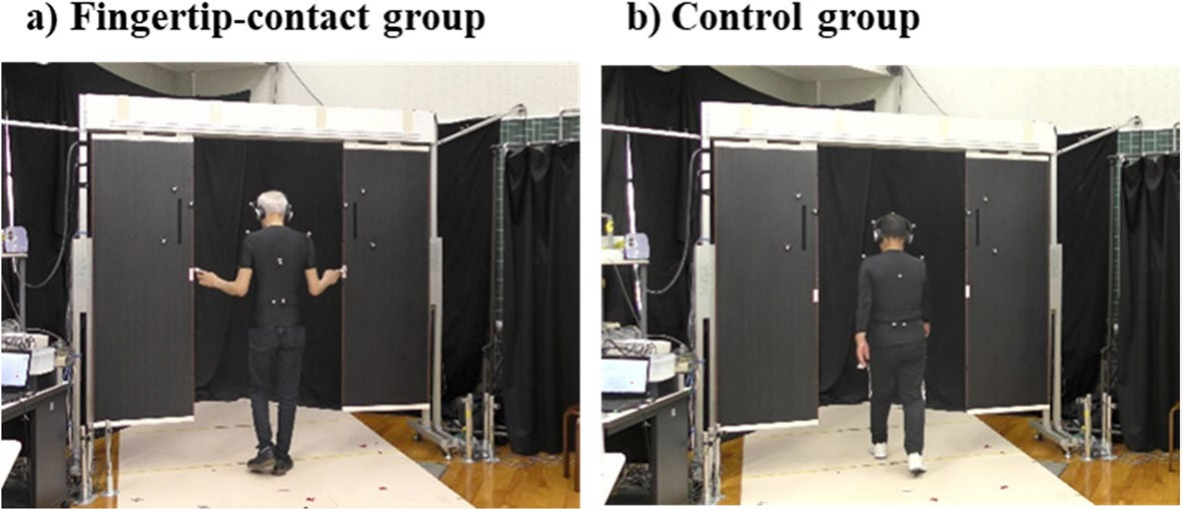
Intervention in the **a** fingertip-contact group and **b** control group in an intervention session

As in the pre and posttests, participants walked and crossed the aperture during the intervention sessions. However, unlike the walking in the pre- and posttests, they did not hold the horizontal bar. Participants in the fingertip-contact group initiated their walking, while both index fingers were held in the extended position with the upper limbs abducted (Fig. 3a). They were asked to walk through apertures of various widths while touching the red tape attached to each side of the door with the tips of their index fingers. They were instructed not to stop or suddenly slow down when directly in front of the aperture in an effort to touch the tape accurately. Participants in the control group walked normally and crossed the aperture (Fig. 3b). As with the fingertip-contact group, they were also instructed not to stop or suddenly slow down when directly in front of the aperture. However, they were not instructed to walk through the aperture with a minimal spatial margin. The aperture widths were the same as those presented in the pre- and posttests (i.e., 0.9, 1.0, and 1.1 times the bar length). The postural requirement of touching the door with both hands resulted in a wide aperture width, and the task was performed without trunk rotation. Participants performed 24 main trials (three aperture widths × eight trials). Three practice trials (three aperture widths × one trial) were performed prior to the main trials.

### Data analyses

To test participant homogeneity between the two experimental groups, the Mann–Whitney *U*-test was performed to statistically compare all participant details (age, height, shoulder width, MMSE score, and TUG test score), excluding gender. A Pearson’s chi-squared test was conducted to identify the gender ratio.

To determine whether collision-avoidance behavior was improved in terms of efficiency after intervention, two dependent variables obtained from pre- and posttest performances were compared statistically.

The first variable was the spatial margin at the time of aperture crossing during successful trials (i.e., trials where no collision occurred). The spatial margin was calculated as the distance between the position of the marker on the index finger (virtual indication of the position of the bar edge) and the inner edge of the board. The time of aperture crossing was defined as the time one of the two markers on the index fingers crossed the aperture first. The values were averaged over the successful trials for each participant and statistically compared using a two-way (group × test) ANOVA with repeated measures test.

The second variable was the collision rate. The collision rate during each test (out of a total of six trials) was calculated for each participant. Given that the error-rate distribution was not normal, we adjusted it using the arcsine transformation for the statistical analysis of a two-way (group × test) analysis of variance (ANOVA). All statistical analyses were conducted using RStudio software (version 2022.02.3).

Several kinematic variables obtained during the pre- and posttests were also analyzed. The mean movement speed at the time of aperture crossing during successful trials was measured, based on the reflection marker placed at the top of the overhead headphones. Measuring the movement speed was necessary to determine whether the improvement in efficient behavior would be accompanied by reduced movement speed (i.e., the speed-accuracy tradeoff). The mean absolute angle of head rotation in the yaw dimension, calculated using left and right markers on the overhead headphones, was measured at the time of aperture crossing during successful trials to address whether the finger-tip contact intervention would affect the head rotation behavior of looking at either side of the space to avoid collision. Both variables were analyzed statistically with a two-way (group × test) analysis of variance (ANOVA). Other kinematic measurements of the reflective markers placed on the body were not used for achieving the purpose of the study and are not reported here.

## Results

### Participants’ characteristics

Participants’ characteristics are summarized in Table 1. No significant main effects between the two groups were found in any of the measurements.

**Table 1:** Participants’ details in each experimental group

	Fingertip-contact group (*n* = 14)	Control group (*n* = 13)	*P*-value
Gender (male/female)^a^	7/7	5/8	0.54
Age (y)^b^	73.6.1 ± 5.1	73.9 ± 4.7	0.79
Height (cm)^b^	160.9 ± 9.2	158.1 ± 9.6	0.48
Shoulder width (cm)^b^	39.9 ± 4.4	40.7 ± 3.2	0.58
MMSE (points)^b^	28.3 ± 1.6	28.3 ± 2.2	0.65
TUG (s)^b^	6.5 ± 0.7	6.4 ± 1.3	0.79

^a^Pearson’s chi-square test

^b^Mann–Whitney *U*-test

### Spatial margin

**Fig. 4:**
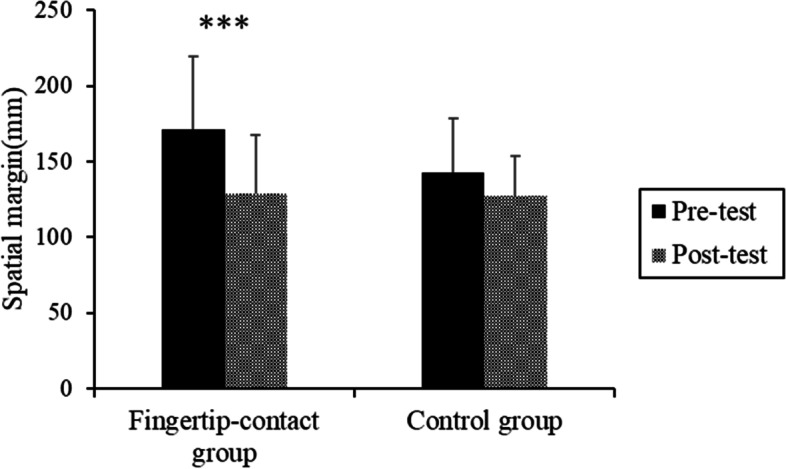
The mean spatial margin in each group

The mean spatial margin in each experimental condition is shown in Fig. 4. An ANOVA showed the main effect of the test (*F* (1, 25) = 28.809, *p* < 0.001, *η*_*p*_^2^ = 0.131). The main effect of the group was not significant (*F* (1, 25) = 1.197, *p* = 0.284, *η*_*p*_^2^ = 0.039, *ns*). A significant interaction was found (*F* (1, 25) = 6.265, *p* = 0.019, *η*_*p*_^2^ = 0.032). In the posttest, the spatial margin for the fingertip-contact group was significantly smaller in the posttest than in the pretest (*F* (1, 50) = 31.617, *p* < 0.001, *η*_*p*_^2^ = 0.207).

### Collision rate

**Fig. 5:**
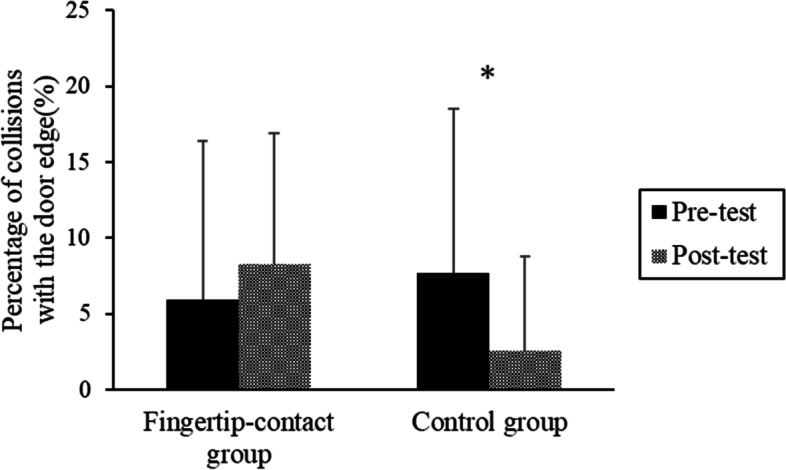
The mean rate of collision with the door in each group

The mean collision rate in each experimental condition is shown in Fig. 5. An ANOVA for the adjusted data using the arcsine transformation showed no main effect of the test (*F* (1, 25) = 0.583, *p* = 0.452, *η*_*p*_^2^ = 0.007, *ns*). The main effect of the group was not significant (*F* (1, 25) = 0.260, *p* = 0.615, *η*_*p*_^2^ = 0.007, *ns*). A significant interaction was found (*F* (1, 25) = 4.36, *p* = 0.047, *η*_*p*_^2^ = 0.051). In the posttest, using Holm corrections, the collision rate of the control group was significantly smaller in the posttest than in pretest (*F* (1, 25) = 5.333, *p* = 0.040, *η*_*p*_^2^ = 0.111).

### Kinematic measurements

The mean movement speed and the head-rotation angle at the time of aperture crossing in each experimental condition are shown in Table 2. For the movement speed, neither the main effect of the test (*F* (1, 25) = 0.508, *p* = 0.482, *η*_*p*_^2^ = 0.02, *ns*) nor that of the group (*F* (1, 25) = 2.055, *p* = 0.164, *η*_*p*_^2^ = 0.076, *ns*) was significant. No significant interaction was found (*F* (1, 25) = 0.066, *p* = 0.799, *η*_*p*_^2^ = 0.003, *ns*). For the absolute angle of head rotation for each group, the main effect of the test was not significant (*F* (1, 25) = 3.118, *p* = 0.090, *η*_*p*_^2^ = 0.111, *ns*). The main effect of the group was significant (*F* (1, 25) = 7.260, *p* < 0.05, *η*_*p*_^2^ = 0.225). No significant interaction was found between the two factors (*F* (1, 25) = 1.758, *p* = 0.197, *η*_*p*_^2^ = 0.066, *ns*).

**Table 2:** Mean movement speed and absolute angle of head rotation in each experimental condition

	Fingertip-contact group		Control group	
	Pretest	Posttest	Pretest	Posttest
Movement speed (cm/s)	91.0 (19.5)	88.0 (19.6)	100.4 (22.7)	99.0 (18.7)
Head rotation angle (deg)	19.3 (9.4)	18.6 (9.6)	13.2 (7.7)	9.2 (5.2)

## Discussion

### Spatial margin

The fingertip-contact group showed a significant decrease in the spatial margin in the aperture-passing task after intervention. This result agreed with the hypothesis. From a reductive view, the spatial relationship between the fingertip and the door was learned based on the error input [17, 18]. It is supposed that repeated adjustment of movements using such haptic feedback information updates the internal model to cope with the discrepancy between the target and actual situations [26]. Notably, an analysis of movement speed showed no significant decrease from the pre- to the posttests in the intervention group. This suggests that the improvement in efficient behavior observed in the post test was not simply derived from decreased movement speed (i.e., the speed–accuracy tradeoff). Analysis of the head-rotation angle showed that although the main effect of the test was significant, the head-rotation angle remained unchanged between the pre- and posttests in the intervention group (Table 2). The head rotation in the yaw dimension at this moment was considered to be related to looking at either side of the space to avoid collision. The results indicate that improvement in efficient behavior did not result from a change in strategy of looking at the space. Rather, the improvement in the efficient behavior may have resulted from improved perception of the body-environment relationship based on the haptic information.

From an emergent view, it is supposed that the task/goal (i.e., perceptual learning of the body-environment relationship) was achieved by the repeated perception-action cycle through haptic feedback, and a transfer of learning might occur from the intervention task (i.e., fingertip-contact task) of the test task (i.e., walking through an aperture with a minimal margin). Considering these theoretical interpretations, the proposed intervention might promote the perception of a spatial relationship between the body and the environment and improve obstacle-avoidance behavior in terms of an efficiency-based strategy.

There was no significant difference between pre- and posttests for the control group. This suggests that intervention without any haptic feedback cannot decrease a participant’s spatial margin when passing the narrow aperture. Such intervention might not improve obstacle avoidance in terms of an efficiency-based strategy.

These results indicated that the proposed intervention (i.e., fingertip contact) can decrease the spatial margin between the body and the environment by learning based on perceptual feedback using haptic information and can improve obstacle-avoidance behavior in terms of motor efficiency. We speculate that such intervention can suppress the excessive avoidance behavior that leads to the balance instability often observed in older adults [6, 7].

### Collision rate

For the fingertip-contact group, there was no significant improvement in the collision rate. These results indicate that touching the obstacle with the fingertips, while reducing the spatial margin to the door, does not necessarily contribute to decreasing collision rates. Three reasons are considered as to why the collision rate did not change after the fingertip-contact intervention. First, as mentioned in the introduction, an efficiency-based strategy can lead to an increased collision risk because participants get close to the obstacle when passing the aperture to keep the margin small. As a result, considering the trade-off relationship between safety and efficiency [27], the collision rate did not decrease, while the spatial margin was reduced in the fingertip-contact group.

Second, participants may have been able to recognize from the haptic information of their fingertips that the risk of collision with the door was low by lightly touching the door. A previous study showed that haptic/tactile information from the fingertips is important for perceiving the material of an object [28]. The door used in this study was equipped with a shock-reducing mechanism, so the risk of injury was extremely low even if participants collided with the door. As a result of perceiving the material of the door from the haptic information of the fingertips, it can be inferred that participants were less afraid of colliding with the door.

Third, there were differences between the test and intervention tasks. The test task required participants to avoid contact with the door. In this case, it is known that participants tend to rotate their body several meters before reaching the door to avoid collision [20, 29]. Thus, participants penetrated and passed the door from either the left or right side of the body by rotating the body (Fig. 2). Contrarily, the intervention task of the fingertip-contact group required participants to touch the door. In this case, participants penetrated and touched the door without rotating their body (Fig. 3). As a result, the test and intervention tasks have different task demands and behavioral patterns. The differences in these two tasks might cause the difficulty in decreasing the collision rate in the fingertip-contact group.

On the other hand, for the control group, participants were required to avoid contact with the door in both the test and intervention tasks. Furthermore, they could learn repeatedly the spatial relationship between their body and the door visually. The similarity in these task demands and repeated visual leaning might cause learning and improvement in passing the door without collision and decreasing the collision rate.

The present results suggest that the proposed intervention method of fingertip contact possibly improves the efficiency-based strategy in terms of reducing the spatial margin and keeping a minimal margin. According to the trade-off relationship between safety and efficiency [27], the collision rate or risk might not always decrease. However, it is important to keep an optimal margin considering both safety and efficiency in obstacle-avoidance tasks. According to previous studies, the excessive avoidance behavior involved in the safety-based strategy can lead to the destabilization of balance during obstacle avoidance in older people [6, 7]. Thus, not only the safety-based strategy but also the efficiency-based strategy is important for finding the optimal spatial relationship between the body and the environment. To do so, the proposed intervention of touching the environment might be useful and meaningful for learning the body-environment relationship. Moreover, learning the optimal body-environment relationship should lead to decreased collision risk and the prevention of falls in older people. Furthermore, there is potential for new interventions incorporating tactile feedback not only in aperture passage but also in training for the avoidance of various obstacles. For example, a target object could be placed above the step (typical foot-lift height) during obstacle avoidance, and touching it would provide feedback regarding the height of the foot lift.

This study has several limitations. First, the long-term learning or retention effect of the proposed intervention should be examined in a future study. Second, it should be investigated whether the excessive avoidance behaviors of older adults can be suppressed by the proposed intervention in terms of efficiency-based strategy. Third, the present study found that participants transferred the behavior learned with the fingertip-contact intervention to walking while holding the horizontal bar. The manipulation of holding the bar was necessary to increase task difficulty during pre- and post-test measurements. However, it limited the natural arm swing during walking. Therefore, it was not certain whether the learned behavior would also be transferred to normal walking. Finally, how this intervention can lead to preventing falls in older adults should be also surveyed in the future.

## Conclusion

The present study revealed that the spatial margin between the body and an obstacle when walking through a narrow aperture immediately decreased after fingertip-contact intervention in healthy older adults, although a possible side effect of increased collision rates was also found. This result suggests that fingertip-contact intervention can improve obstacle-avoidance behavior with a minimal margin (i.e., efficiency-based strategy). Although some older adults tend to show excessive obstacle-avoidance behavior by keeping a larger spatial margin, which can cause balance instability, the proposed intervention might promote an efficiency-based strategy due to learning the optimal spatial relationship between the body and the environment and, thus, suppress excessive avoidance. Further investigation is needed to reveal whether such a proposed intervention can prevent falls by older adults in the future.

## Data Availability

The datasets during and/or analyzed during the current study are available from the corresponding author upon reasonable request.

## Abbreviations

MMSE: Mini-Mental State Examination
TUG: Timed Up and Go test
ANOVA: Analysis of variance
